# Transcutaneous Vagal Nerve Stimulation Alone or in Combination With Radiotherapy Stimulates Lung Tumor Infiltrating Lymphocytes But Fails to Suppress Tumor Growth

**DOI:** 10.3389/fimmu.2021.772555

**Published:** 2021-12-01

**Authors:** Eva Reijmen, Sven De Mey, Helena Van Damme, Kirsten De Ridder, Thierry Gevaert, Emmy De Blay, Luc Bouwens, Christine Collen, Lore Decoster, Marijke De Couck, Damya Laoui, Jacques De Grève, Mark De Ridder, Yori Gidron, Cleo Goyvaerts

**Affiliations:** ^1^ Laboratory for Molecular and Cellular Therapy, Department of Biomedical Sciences, Vrije Universiteit Brussel, Brussels, Belgium; ^2^ Department of Radiotherapy, Oncology Centre University Hospital Brussels (Universitair Ziekenhuis (UZ) Brussel), Brussels, Belgium; ^3^ Myeloid Cell Immunology Lab, Vlaams Instituut voor Biotechnologie (VIB) Center for Inflammation Research, Brussels, Belgium; ^4^ Lab of Cellular and Molecular Immunology, Vrije Universiteit Brussel, Brussels, Belgium; ^5^ Cell Differentiation Lab, Vrije Universiteit Brussel, Brussels, Belgium; ^6^ Laboratory of Medical and Molecular Oncology (LMMO), Department of Medical Oncology, Oncologisch Centrum, Universitair Ziekenhuis (UZ) Brussel, Vrije Universiteit Brussel (VUB), Brussels, Belgium; ^7^ Department of Public Health, Mental Health and Wellbeing Research Group, Faculty of Medicine and Pharmacy, Vrije Universiteit Brussel, Brussels, Belgium; ^8^ Faculty of Health Care, University College Odisee, Aalst, Belgium; ^9^ Faculty of Social Welfare and Health Sciences, University of Haifa, Haifa, Israel

**Keywords:** neuromodulation, transcutaneous vagal nerve stimulation, radiotherapy, immunosuppressive tumor microenvironment (TME), lung cancer, tumor infiltrating lymphocytes

## Abstract

The combination of radiotherapy (RT) with immunotherapy represents a promising treatment modality for non-small cell lung cancer (NSCLC) patients. As only a minority of patients shows a persistent response today, a spacious optimization window remains to be explored. Previously we showed that fractionated RT can induce a local immunosuppressive profile. Based on the evolving concept of an immunomodulatory role for vagal nerve stimulation (VNS), we tested its therapeutic and immunological effects alone and in combination with fractionated RT in a preclinical-translational study. Lewis lung carcinoma-bearing C57Bl/6 mice were treated with VNS, fractionated RT or the combination while a patient cohort with locally advanced NSCLC receiving concurrent radiochemotherapy (ccRTCT) was enrolled in a clinical trial to receive either sham or effective VNS daily during their 6 weeks of ccRTCT treatment. Preclinically, VNS alone or with RT showed no therapeutic effect yet VNS alone significantly enhanced the activation profile of intratumoral CD8^+^ T cells by upregulating their IFN-γ and CD137 expression. In the periphery, VNS reduced the RT-mediated rise of splenic, but not blood-derived, regulatory T cells (Treg) and monocytes. In accordance, the serological levels of protumoral CXCL5 next to two Treg-attracting chemokines CCL1 and CCL22 were reduced upon VNS monotherapy. In line with our preclinical findings on the lack of immunological changes in blood circulating immune cells upon VNS, immune monitoring of the peripheral blood of VNS treated NSCLC patients (n=7) did not show any significant changes compared to ccRTCT alone. As our preclinical data do suggest that VNS intensifies the stimulatory profile of the tumor infiltrated CD8^+^ T cells, this favors further research into non-invasive VNS to optimize current response rates to RT-immunotherapy in lung cancer patients.

## Introduction

Radiotherapy (RT) has an important and established role in the treatment of patients with non-small cell lung cancer (NSCLC). However, for patients with inoperable stage III disease, outcomes after conventional RT (typically 1.8-2.0 Gy per day for a total dose of 60-70 Gy) are modest with a 5-year survival of less than 15% (1). In the past decade, exponential developments in the field of immunotherapy have profoundly improved the prognostic algorithm. Today, advanced NSCLC patients can cherish the hope that they remain progression-free for several years after diagnosis upon treatment with immune checkpoint inhibitors (ICI) that target Programmed Death-1 (PD-1) or its ligand (PD-L1). Yet, this is only the case for 20-25% of patients (2, 3). These sobering numbers suggest that additional immunomodulatory pathways need to be explored to harness at maximum the effects of radio(chemo)therapy and ICI, alone or in combination.

Immunotherapy barged into the treatment arsenal for cancer because cancer progression has been found to be profoundly shaped by the host innate and adaptive immune system. The so-called cancer immune-editing process results in a tumor-immune battlefield with three phases: tumor elimination, equilibrium and escape (4, 5). During the tumor elimination phase, the innate immunity arm exploits myeloid and lymphoid effector cells like macrophages and natural killer (NK) cells respectively (6). In addition, mutations can result in expression of tumor associated antigens (TAAs) that enable the adaptive immune system to distinguish healthy from malignant self. Specifically, immature dendritic cells (DCs) can capture TAAs that are released from dying cancer cells (7, 8), which are subsequently processed and presented *via* major histocompatibility complex (MHC) molecules to naïve T cells. This can trigger a protective T-cell mediated response composed of TAA-specific CD4^+^ helper and CD8^+^ cytotoxic T lymphocytes (CTL). Hence, both tumor mutational burden and tumor infiltrating lymphocytes have been shown to correlate with a better overall and progression-free survival in patients with NSCLC (9, 10). However, tumor cells can escape this elimination phase *via* numerous ingenious mechanisms, often characterized by the installation of an immunosuppressive tumor microenvironment (TME) (11). In progressing lung cancers, this TME has been characterized by the exclusion of cytolytic NK cells next to an enrichment for non-functional (granzyme B^-^) T cells and Foxp3^+^ regulatory T cells (Tregs). The latter can further suppress the cytolytic effector functions of CTLs and NK cells by producing immunosuppressive cytokines (e.g. IL-10 and TGF-β), expressing immune suppressive checkpoints (e.g. PD-1 and PD-L1) and consuming IL-2, critical for maintaining CTL function (7, 11, 12). The myeloid compartment is mainly represented by CD1c^+^ conventional type 2 DCs, M2-like tumor associated macrophages (TAMs), suppressive monocytes and granulocytes (collectively termed MDSCs) (13), that can further mold the TME into a CTL-hostile milieu (7, 11, 14–16). Importantly, the frequency of MDSCs has been negatively correlated to therapeutic efficacy of RT, chemotherapy and ICIs (17–19).

While tumor irradiation can activate anti-tumor immunity through the induction of immunogenic cell death, the latter can also be counteracted by the accumulation of radioresistant immunosuppressive cells, including TAMs, MDSCs and Tregs (20, 21). We previously confirmed that systemic and especially local immunosuppression predominates in a murine orthotopic NSCLC model when treated with clinically relevant, low-dose fractionated RT (22). In addition to reduced numbers of CTLs and mature CD86^+^ DCs, the fractions of TAMs, Tregs, monocytes and neutrophils increased upon RT treatment. These findings suggest that therapeutic strategies combining RT with ICI may benefit from combination strategies that curtail the immunosuppressive myeloid compartment and reinforce T-cell immunosurveillance.

A newly identified mechanism of immunosuppression is sympathetic adrenergic signaling with repercussions for the development, differentiation, activation and function of various immune cell types (23–26). Several studies in mice and humans reinforce growing recognition of a negative role of the sympathetic nervous system (SNS) response in cancer progression, metastasis and treatment resistance (27–32). Parasympathetic/vagal activity generally antagonizes the effects of the SNS (33) and research in the last decade revealed that the vagal nerve (VN) is an immunomodulator (34). Moreover, in several experimental models of inflammatory disease, VN stimulation (VNS) is shown to attenuate the production of pro-inflammatory cytokines and inhibit inflammatory processes through the so-called ‘cholinergic anti-inflammatory pathway’ (35–37). Moreover, experimental murine studies demonstrated that the vagal pathway withholds widespread epigenetic and immunologic influence with presumptive anti-tumoral effects (38–42). Surgical or pharmaceutical removal of VN activity (vagotomy) has been shown to increase lung, liver and kidney metastasis of breast cancer cells in mice (43, 44) whereas VNS reduced distant metastasis of breast cancer cells (45). Also, epidemiological studies showed that high vagal activity, indexed by heart-rate variability, predicted longer survival in colon cancer, prostate cancer, breast cancer and NSCLC (46–48). Hence, the aim of the present preclinical-translational study was to investigate the hypothesis that transcutaneous VNS can reduce RT-mediated provocation of an immunosuppressive lung TME. Our data support a potentially immunostimulatory effect of VNS and the possibility that VNS could help overcome RT-induced immunosuppression.

## Materials and Methods

### Experimental Mouse Model

#### Cells

Lewis lung carcinoma, positive for Firefly luciferase (LLC-Fluc), were previously generated using a Fluc-encoding lentiviral vector (transfer plasmid pDUAL_SFFV-Fluc_Ub-puroR) as described (22, 49). Cells were maintained in Dulbecco’s Modified Eagle’s Medium (DMEM, Sigma-Aldrich) supplemented with 10% fetal bovine serum (FBS, Harlan), 100 units/ml penicillin, 100 µg/ml streptomycin and 2 mM L-Glutamine (Sigma-Aldrich) at 37°C, 5% CO_2_, 21% O_2_ and humidity level of 95%. Prior to their intravenous (i.v.) injection, LLC-Fluc cells were subjected to one round of puromycin selection (1μg/ml for 3 days) to enrich the Fluc positive fraction.

#### Mice

Six- to eight-week-old female C57BL/6 mice were purchased from Charles River (L’Abresle, France) and maintained in our animal facility under pathogen-free conditions. All animal protocols were in accordance with the European guidelines for animal experimentation and authorized by the Ethical Committee for Laboratory Animals of the Vrije Universiteit Brussel (ethical dossier numbers: 18-281-8 and 20-214-14). To obtain lung tumor bearing mice (n=46), 5 x 10^5^ LLC-Fluc cells dissolved in 200µL phosphate buffered saline (PBS, Sigma-Aldrich), were injected i.v.

### Patient Population

Patients diagnosed with locally advanced NSCLC receiving standard-of-care radiation therapy with concurrent chemotherapy (ccRTCT) were eligible for enrollment in the study. Patients were excluded if they had any of the following contraindications: recent (<6 months) stroke or myocardial infarction, severe heart failure (class III or IV), patients with an active implanted medical device (pacemaker, defibrillator or hearing aid implant), recurrent vasovagal syncope, unilateral or bilateral vagotomy, sick sinus syndrome (without a pacemaker), 2nd or 3rd-degree atrioventricular block and pregnancy or nursing. There were no differences between the two treatment groups in regards to age, tumor size, BMI and smoking history. The clinical study was approved by the local medical ethical committee (2018/016) and registered on ClinicalTrials.gov (identifier: NCT03553485). All patients signed informed consent before inclusion in the study.

### Lung Tumor Treatment Regimens

#### Mice

One week after LLC-Fluc injection, *in vivo* bioluminescence imaging (BLI) was used to visualize tumor burden (50) and randomize mice with similar photon counts to one of four different treatment groups: VNS only, RT+sham, RT+VNS or sham (control). Radiation treatment was performed from day 11 in 4 consecutive daily fractions of 3,2 Gy as previously described (22). In brief, Ketamine (Ketamidor^®^, UK, 100mg kg ^-1^)/Xylazine (Rompun^®^, Germany, 10 mg kg ^-1^) anesthetized mice were positioned in a 3D-printed mold (Ultimaker Extended 2+ using PLA filaments) and irradiated with the indicated doses using the Truebeam STx system (Varian, Palo Alto, CA, USA; BrainLAB AG, Feldkirchen, Germany). VNS treatment was performed from day 11 until day 14 using the non-invasive stimulator ‘gammaCore’ (electroCore, LLC, Basking Ridge, NJ, USA) developed for mice. Prior to stimulation, a conducting gel (Signa gel, Parker Laboratories, Fairfield NJ) was applied to surfaces of the disc electrodes which were then placed on the shaved neck of the mouse lateral to the trachea and over the left cervical vagus nerve. Electrical stimulation (1 msec duration, 5 kHz, 12 V sine waves repeated at 25 Hz; impedance: 350 ohm) was delivered twice per day in the form of 3 successive 2-minute trains.

#### Patients

All patients received cisplatin plus docetaxel weekly and concomitant standard RT for a total of 67,2 Gy at 2,24 Gy/fraction/day 5 times weekly for 6 weeks. VNS treatment consisted of two separate, 30-minute sessions of active or sham VNS daily during their course of ccRTCT. Stimulation was provided using a transcutaneous vagal nerve stimulation (tVNS) device, consisting of a pulse generator and electrode targeting the auricular branch of the vagal nerve *via* the external ear (Parasym device, Parasym Ltd, London, UK). The tVNS device was attached to the ear tragus and set at a pulse width of 200μs and pulse frequency of 25 Hz in the active group. The stimulation amplitude was individualized to 1mA below the discomfort threshold. Sham treatment did not deliver stimulation as electrical wiring in the electrode was removed. The choice of the VNS stimulation parameters applied was based on previous reports using the GammaCore and Parasym VNS devices. It is important to note, however, that as a proof-of-concept study, the present work was not designed to evaluate different stimulation parameters.

### Preparation of Single Cell Suspensions From Murine Blood, Lung, and Spleen

All mice underwent submandibular blood sampling prior to (D_7_), during (D_13_) and after completion (D_20_) of their treatments. Blood (200μl) was collected in heparin-coated tubes (Sarstedt, Germany), centrifuged for 10 minutes at 2000g to separate blood cell pellets from plasma. While cell pellets were immediately analyzed, plasma samples were stored at -20°C for further analysis. At day 20 after tumor injection, mice were sacrificed by cervical dislocation and single cell suspensions from lung and spleen were prepared. Lungs were first perfused with 5ml PBS and transferred to 1ml Roswell Park Memorial Institute-1640 medium (RPMI-1640, Sigma-Aldrich) containing 300U/ml collagenase-I (Sigma-Aldrich). Tissues were cut to small pieces using scissors, incubated at 37°C for 45 minutes, and finally mechanically reduced using an 18G syringe until single cell suspensions could be passed through a 40μm strainer. Spleens were transferred to 1ml PBS, stamped with the plunger of a 3cc syringe and passed through a 40μm strainer. Cell pellets from blood, lung and spleen, were resuspended in 1ml red blood cell lysis buffer, incubated for 5 minutes, followed by a centrifugation and wash step with PBS before further analysis.

### Murine *Ex Vivo* T Cell Restimulation Assay

Cell suspensions from murine lung tissue were cultured at 3 x 10^5^ cells/100µl complete RPMI medium with 30ng/ml recombinant IL-2 (Peprotech) in the presence of 3 x 10^4^ LLC cells. After 24 hours, supernatants were collected for evaluation of IFN-γ secretion *via* a mouse IFN-γ ELISA kit (Invitrogen, in accordance with the manufacturer’s guidelines). Cells were treated for an additional 4 hours with the protein transport inhibitor Golgi-stop (Monensin, BD Biosciences) prior to surface and intracellular staining for CD137, IL-2 and IFN-γ within the CD8^+^ T lymphocytes.

### Isolation and Storage of Human Plasma and PBMCs

Peripheral blood (8ml) was collected in EDTA-coated tubes before (D_0_), during (D_21_) and at the end (D_42_) of the RT regimen. To isolate the peripheral blood mononuclear cells (PBMCs), samples were centrifuged at 2200 rpm for 10 minutes. While plasma was immediately stored at -80°C for further analysis, PBMCs were purified using LeucoSep tubes according to the manufacturers’ instructions (Greiner Bio-One). In brief, cell pellets were diluted with equal volumes of PBS before transfer to LeucoSep tubes and centrifugation at room temperature for 15 min at 800 rcf without brake. Next, the PBMCs were collected, washed twice in PBS and aliquoted before cryopreservation in liquid nitrogen.

### Immunological Evaluation of Murine and Human Plasma

Concentrations of cytokines, chemokines and growth factors were evaluated on 12,5μl of murine plasma sample *via* cytokine bead array technology (BIO-RAD, Bio-Plex 200 System). More specifically the Bio-Plex Pro Mouse Chemokine Panel, 31-plex (BIO-RAD) and a Mouse TGF-β1 ELISA kit (Thermo Fisher Scientific) were used.

Concentrations of human TGF-β1, I-309/CCL1, ENA-78/CXCL5 and MDC/ADAM11 were measured using the respective human ELISA kits (Thermo Fisher Scientific) in accordance with the manufacturer’s instructions.

### Histology

The large left lobe of each murine lung was fixed in buffered 4% formaldehyde and paraffin embedded. Tissue sections of 4μm were deparaffinized, hydrated and stained with haematoxylin, eosin and saffron (HES). The tissue sections were then dehydrated and mounted to allow histological evaluation. Immunohistochemistry images were acquired using a Leica DM 4000 microscope at 20x magnification.

### Flow Cytometry Analysis

Staining of surface markers was performed for 30 minutes at 4°C in cold PBS containing 1% bovine serum albumin (BSA, Sigma-Aldrich) and 0.02% sodium azide (FACS buffer; Sigma-Aldrich). The respective fluorescently labeled antibodies are listed in **Supplementary Tables 1** and **2** (**Supplementary Material**). To avoid non‐specific antibody binding, Fc receptors were first blocked by incubating all samples with CD16/32 antibody (BD Biosciences). For intracellular staining, cells were subsequently fixed and permeabilized using Cytofix/Cytoperm™ kit (BD Biosciences). Therefore, cells were washed using Perm/Wash buffer, resuspended in Fixation/Permeabilization solution for 20 minutes at 4°C. Next, samples were washed twice using the Perm/Wash buffer prior to staining with the anti-arginase-1-PE antibody (R&D systems, Abingdon, United Kingdom), diluted in Perm/Wash buffer for 30 minutes at 4°C. The stained cells were evaluated on an LSRFortessa flow cytometer (Beckton Dickinson) while analysis was performed with the FlowJo 10.5.3 software (Tree Star Inc., Ashland, OR, USA).

### Statistical Methods

Two-tailed unpaired t-tests were used to determine the significance of differences. A P value ≤ 0.05 was used as cut-off value for significance. Aggregated data are presented in figures using mean values to represent the central tendency and standard error of the mean (SEM) to represent variability. All statistical analysis was computed using GraphPad Prism v 7.0.

## Results

### VNS Monotherapy Promotes a Local and Peripheral Anti-Tumor Immune Profile

**Figure 1:**
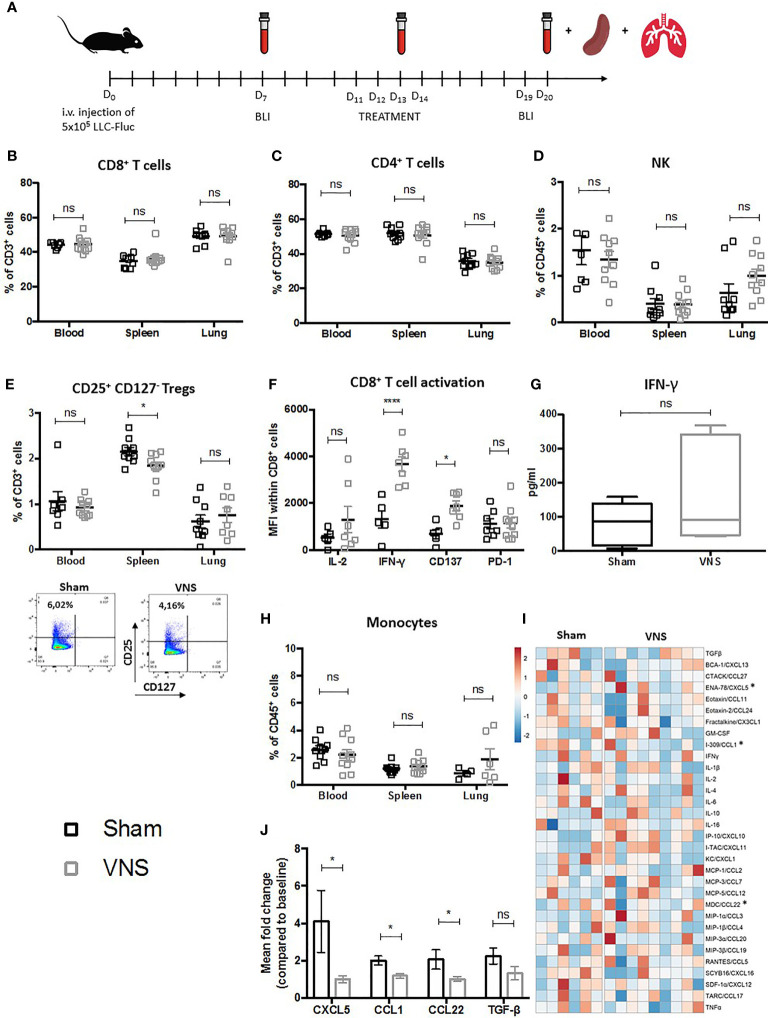
Schematic representation of preclinical experimental setup and evaluation of immune cell populations in lung tumor bearing mice treated with VNS. **(A)** C57BL/6 mice were i.v. injected with 5x10^5^ Fluc expressing LLC tumor cells. Seven days later *in vivo* BLI was performed to randomize mice with similar photon counts to the VNS treated, RT treated, RT+VNS treated or sham treated control group. Mice were daily treated with VNS (25Hz) and/or RT (3,2 Gy) for four consecutive days starting at day eleven after tumor injection. Blood samples were collected at day 7 (baseline), day 13 and day 20. On day 20 after tumor injection, mice were euthanized to collect perfused lungs and spleens. **(B–E, H)** Flow cytometric analysis of **(B)** CD8^+^ T cells, **(C)** CD4^+^ T cells, **(D)** CD56^+^ NK cells, **(E)** CD4^+^CD25^+^CD127^-^ Tregs with on the right a representative dot plot from splenic CD4^+^CD25^+^CD127^-^ Tregs from a sham or VNS treated animal and **(H)** CD11b^+^Ly6C^+^ monocytes in blood, spleen and lung TME. **(F, G)** Cell suspensions derived from murine lung tissue were co-cultured with LLC cells at a 10:1 ratio for 24 hours. Next, expression of IL-2, IFN-γ, CD137 (4-1BB) and PD-1 was assessed on the CD8^+^ T cells specifically using flow cytometry **(F)** and IFN-γ secretion was evaluated *via* a mouse IFN-γ ELISA kit **(G)**. **(I, J)** Data obtained from a 31-plex cytokine array on plasma samples obtained from VNS or sham treated mice. **(I)** Heatmap of the 31 different cytokines, in which the meanfold change of each cytokine concentration compared to baseline is visually represented with red, white, and blue to indicate high, median, and low, respectively. **(J)** Box plots show the significant fold changes of ENA-78 (CXCL5), I-309 (CCL1), MDC (CCL22) next to the non-significant decrease of TGF-β in the same plasma samples. Data represent three pooled experiments six days after the last treatment. Data are shown as mean ± SD of n>9 (VNS) and n>6 (sham). *P < 0,0332; ****0,0001 by two-tailed unpaired t-tests. ns, non significant.

The delicate balance between immune stimulatory effector cells and pro-tumoral suppressive immunocytes represents a critical determinant for lung cancer patient prognosis. Therefore, we first investigated the impact of VNS monotherapy on this balance within the lung TME and periphery (blood and spleen) of lung-tumor-bearing mice. More specifically, 11 days after C57BL/6 mice were challenged i.v. with LLC-Fluc cells, VNS (25Hz) or sham treatment was executed twice daily, for 4 consecutive days (**Figure 1A**). Blood samples were collected at D_7_ (baseline), 3 days after the first (D_13_) and 6 days after the last (D_20_) treatment, prior to euthanization. Aside from blood at D_20_, perfused lungs and spleens were also collected to evaluate the following immune fractions in flow cytometry: CD8^+^ CTLs, CD4^+^ T cells, CD19^+^ B cells, CD56^+^ NK cells, CD11c^+^ DCs, SiglecF^+^ alveolar macrophages (AMs), F4/80^+^ TAMs, CD25^+^/CD127^-^ Tregs, Ly6G^+^ neutrophils and Ly6C^+^ monocytes. Gating strategies of lymphoid and myeloid cell populations are provided as **Supplementary Material** (**Figure S1**).

No marked lymphoid differences were observed for the fractions of CD8^+^ CTLs (**Figure 1B**), CD4^+^ helper T cells (**Figure 1C**) between sham and VNS treated animals. Interestingly, VNS did result in a non-significant trend towards more NK cells in the lung TME (**Figure 1D**) and a significantly reduced percentage of splenic CD4^+^/CD25^+^/CD127^-^ Tregs (**Figure 1E**). To investigate the functional impact of VNS monotherapy on the tumor infiltrated CD8^+^ lymphocytes, we co-cultured lung tumor derived cells from sham and VNS treated animals with LLC cells at ratio 10:1 *in vitro*. Twenty-four hours later, expression of the following functional markers on the CD8^+^ TILs was assessed *via* flow cytometry: CD137 (4-1BB), IL-2, IFN-γ and PD-1. We observed a significant increase of IFN-γ and CD137 in the CTLs derived from VNS treated mice (**Figure 1F**). Moreover, within the supernatants a confirmative -yet not significant- trend for enhanced IFN-γ secretion upon VNS was shown by ELISA (**Figure 1G**), suggesting that VNS treatment ameliorated the cytotoxic profile of lung tumor infiltrated CTLs.

When we assessed changes in the myeloid compartment of blood, spleen and lung, VNS treatment did not seem to result in any significant changes in the abundance of DCs, AMs, TAMs nor neutrophils (data not shown) except for a non-significant trend towards more monocytes in the lung TME (**Figure 1H**). To further decipher the systemic immunomodulatory effects of VNS, we analyzed plasma levels of 32 cytokines and chemokines (**Figures 1I, J**). As depicted in **Figure 1J**, VNS treatment resulted in a significant reduction of I-309/CCL1 and MDC/CCL22, which is in line with peripheral reduction of Tregs, since both have been described to regulate Treg recruitment in murine cancer models (51–55). Although we did not see any changes in the neutrophilic fraction, multiplex analysis did show a significant reduction in the plasma levels of the neutrophil-attracting chemokine ENA-78/CXCL5 (**Figure 1J**). In addition, there was a non-significant trend towards reduced plasma levels of TGF-β, renowned for its negative effect on anti-tumor immunity by suppressing immune effector cell functions of, amongst others, CTLs, CD4^+^ T cells and NK cells while promoting the generation and recruitment of Tregs (56–61).

### VNS Specifically Enhances RT-Mediated Immune Stimulation of Lung Infiltrated CTLs

**Figure 2:**
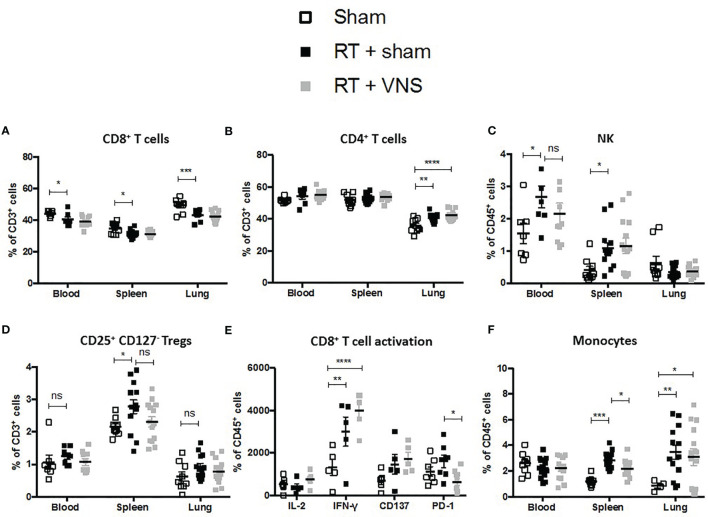
Evaluation of immune cell populations in lung tumor bearing mice treated with RT+VNS. Flow cytometric analysis of lymphoid **(A)** CD8^+^ T cells, **(B)** CD4^+^ T cells, **(C)** CD56^+^ NK cells, **(D)** CD4^+^CD25^+^CD127^-^ Tregs in blood spleen and lung TME. **(E)** Cell suspensions from murine lung tissue were co-cultured with LLC cells at ratio 10:1 *in vitro*. After 24 hours, CD8^+^ TIL expression of the function markers IL-2, IFN-γ, CD137 (4-1BB) and PD-1 was assessed *via* flow cytometry. **(F)** Flow cytometric analysis of myeloid CD11b^+^Ly6C^+^ monocytes in blood spleen and lung TME. Of note, the sham group in this figure represents the same group as in . Data represent three pooled experiments six days after the last treatment. Data are shown as mean ± SD of n=13 (RT) and n=14 (VNS+RT). *P < 0,0332; **0,0021; ***0,0002; ****0,0001 by two-tailed unpaired t-tests. ns, non significant.

**Figure 3:**
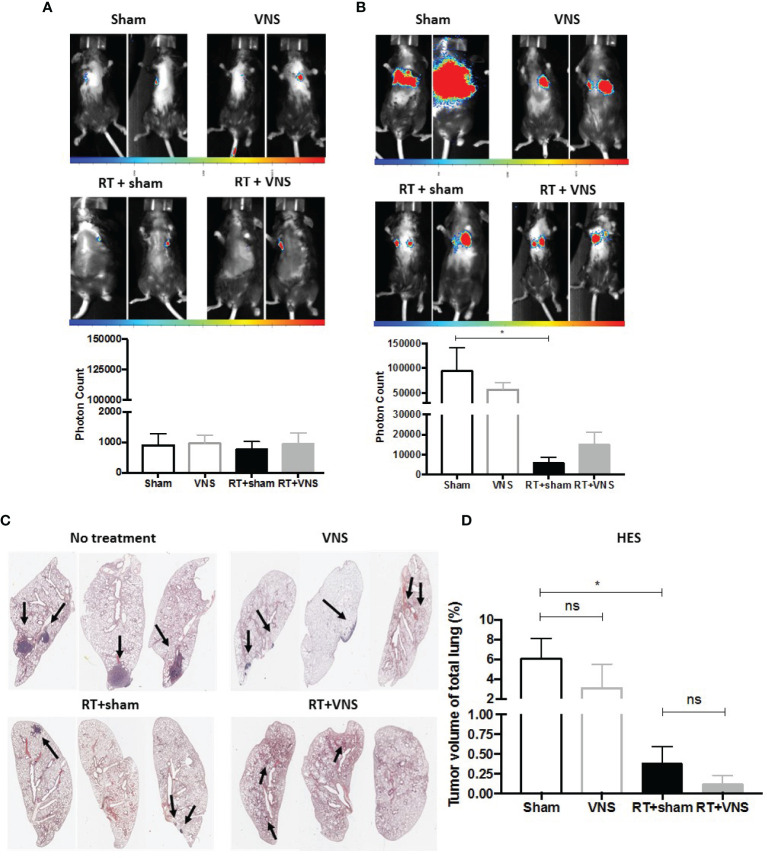
Therapeutic impact of VNS mono- or combitreatment with RT. **(A, B)** On day 7 and 19, lung tumors were evaluated using *in vivo* BLI. Images of two animals from 4 different treatment groups on **(A)** day 7 and **(B)** day 19 with the integrated light signal of 7 minutes at peak activity and photon counts as a measure of tumor size according to ROI. **(C)** Lung histopathology of formalin-fixed, paraffin-embedded lung tissue stained with HES. **(D)** Histology measured nodule volume shown as percentage (%) of total lung volume. Data are shown as mean ± SD of n=4 (sham), n=5 (VNS), n=5 (RT) and n=5 (VNS+RT). *P < 0,0332 by two-tailed unpaired t-tests. ns, non significant.

Using a similar experimental setup and lung tumor model as depicted in **Figure 1A**, we previously observed that fractionated RT results in significant reduction of lung tumor growth (22). However, we and others showed that fractionated RT results in a reduction of the tumor infiltrated CD8^+^ CTL fraction, while the abundance of immunosuppressive myeloid cells increased. To investigate the impact of VNS on these fluctuations, LLC-Fluc bearing mice were treated with RT in combination with sham (four consecutive daily fractions of 3.2 Gy) or in combination with VNS (25Hz) 2 times daily (**Figure 1A**). Flow cytometric analysis of blood, spleen and the lung TME showed that VNS had no impact on the RT-induced reduction of CD8^+^ T cells (**Figure 2A**) nor on the RT-mediated rise in tumor infiltrated CD4^+^ T cells (**Figure 2B**). In addition, no significant changes were found for the numbers of NK cells nor Tregs between mice treated with RT + sham and RT + VNS (**Figures 2C, D**). Notably, VNS did ameliorate the RT-induced increase of IFN-γ^+^ TILs and VNS of RT treated animals further resulted in a significantly reduced expression of PD-1 in the lung TME-residing CTLs (**Figure 2E**). While for the myeloid subsets evaluated in the periphery and lung TME, no significant changes were found, the levels of splenic CD11b^+^ Ly6G^-^ Ly6C^hi^ monocytes did reduce significantly upon RT + VNS compared to RT + sham (**Figure 2F**). Finally, no significant changes in any of the 32 different chemo- and cytokines were found in the plasma of mice treated with RT + sham and RT + VNS (data not shown). To evaluate the therapeutic impact of VNS alone or on RT treatment, orthotopic lung tumor growth was assessed *via* BLI (**Figures 3A, B**) and immunohistochemistry (**Figures 3C, D**). VNS monotherapy as well as in combination with RT did not result in tumor growth reduction compared to sham alone or RT + sham respectively (**Figures 3B, D**).

### VNS of RT Treated NSCLC Patients Slightly Improves the Serological Immune Balance

**Figure 4:**
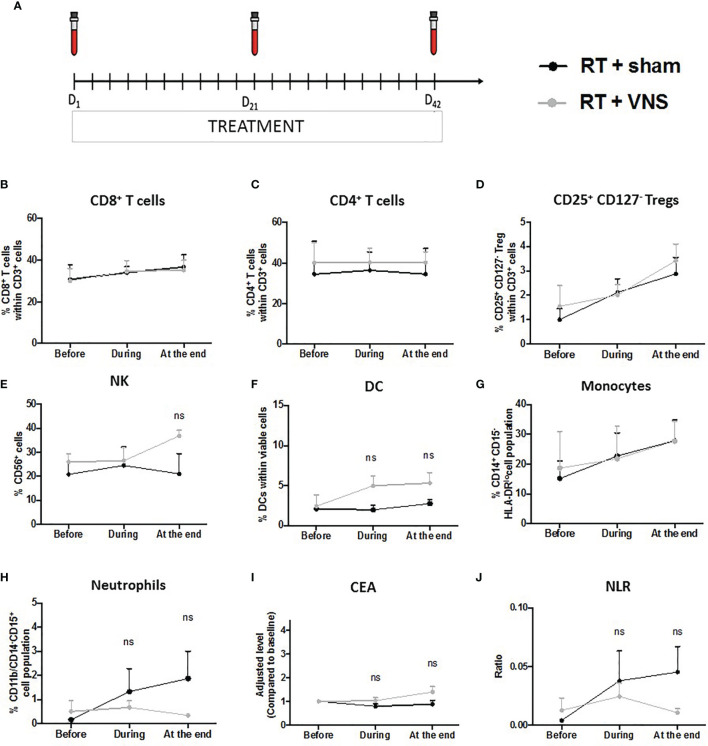
Schematic representation of the clinical trial and impact on lymphoid and myeloid cell populations in NSCLC patients. **(A)** Subjects’ visit schedule. Bloods were drawn before the first VNS treatment was performed (baseline), after 3 weeks of ccRTCT (D_21_) and at the end of ccRTCT (D_42_). Flow cytometric analysis comparing the levels of **(B)** CD8^+^ T cells, **(C)** CD4^+^ T cells, **(D)** CD25^+^CD127^-^ Tregs, **(E)** NK cells, **(F)** DC, **(G)** CD14^+^CD15^−^HLA-DR^lo/–^ monocytes and **(H)** CD11b^+^CD14^−^CD15^+^ neutrophils in both cohorts. Adjusted serum levels (normalized against baseline levels) of the prognostic markers **(I)** CEA and **(J)** NLR. ns, non significant.

To gain more insights into the potential immunomodulatory effect of VNS on RT treated NSCLC patients, we performed a pilot clinical study. Here we analyzed the effect of VNS on the composition of PBMCs in patients with locally advanced NSCLC receiving ccRTCT. We collected blood samples from 7 patients (n=3 standard ccRTCT, n=4 standard ccRTCT + VNS) before (D_1_), during (D_21_) and at the end of their treatment (D_42_) (**Figure 4A**) to analyze cellular and molecular changes. Patients’ and tumor characteristics are summarized in **Table 1**.

**Table 1:** Patients characteristics at baseline.

RT + sham				Mean ± SEM	RT + VNS				Mean ± SEM	P-value
Patient	1	2	3		1	2	3	4		
**Age**	71	58	50	60 ± 6.1	68	56	64	68	64 ± 2.8	0.51
**Gender**	M	F	F		M	F	M	M		
**Tumor Size (mm)**	54	33	41	42.7 ± 6.1	29	37	40	24	40.8 ± 4.1	0.80
**Smoking History (pys)**	75	60	30	55 ± 13.2	30	43	50	40	40.75 ± 4.2	0.29
**TNM stage**	T3N0M0	T4N2M0	T4N0M0		T1N2M0	T4N2M0	T3N0M1b	T1N2M0		
**Histo-logical tumor type**	Adeno-carcinoma	Squamous cell carcinoma	Adeno-carcinoma		Large cell neuro-endocrine carcinoma	Squamous cell carcinoma	Adeno-carcinoma	Adeno-carcinoma		
**BMI**	31	36	23	30 ± 3.8	35	25	37	26	30.8 ± 3.1	0.88

To determine the numbers of circulating T lymphocytes, DCs, NK cells, monocytes (CD14^+^CD15^−^HLA-DR^lo/–^) and neutrophils (CD11b^+^CD14^−^CD15^+^), we performed flow cytometry on freshly isolated blood samples. The gating strategies of the different lymphoid and myeloid cell populations are provided as Supplementary material (**Figure S2**). In line with our preclinical murine findings, the proportions of CD8^+^ and CD4^+^ T cells did not differ between the standard ccRTCT group and the investigational ccRTCT + VNS group (**Figures 4B, C**). While we did find a reduction in splenic Tregs upon VNS treatment of tumor bearing animals, no changes in murine blood Tregs were found, again in line with the lack of changes in blood Tregs upon VNS treatment of NSCLC patients (**Figure 4D**). To test the functionality of CD8^+^ T cells, we determined the expression of CD107a, IFN-γ, Granzyme-B and PD-1, showing no substantial differences between the two treatment groups (data not shown). The number of anti-tumoral NK cells and DCs within the fraction of blood mononuclear cells remained stable during the 6 weeks of ccRTCT while these populations slightly increased in the ccRTCT + VNS group (**Figures 4E, F**). Whereas VNS had systemic immunomodulatory effects on the following 3 murine chemokines, I-309/CCL1, MDC/CCL22 and ENA-78/CXCL5, we found no indication for an effect on their human isoform (data not shown) nor any changes in the fraction of monocytes (**Figure 4G**). Comparing levels of neutrophils, we found a more pronounced increase of neutrophils in the standard treatment group compared to the investigational group (**Figure 4H**). In advanced NSCLC, peripheral blood biomarkers including the tumor marker carcinoembryonic antigen (CEA) and the neutrophil over lymphocyte ratio (NLR) have been proposed as prognostic biomarkers useful for treatment monitoring (62). In particular, higher CEA and NLR have shown a significant association with worse outcomes (15, 62, 63). When serum levels of CEA were extracted from the electronic patient record system, no differences in the frequency between NSCLC patients receiving the conventional treatment compared to the experimental combination treatment were found (**Figure 4I**). While ccRTCT treated patients showed a slight increase in NLR, this level decreased in the ccRTCT + VNS group, suggestive for a trend towards VNS-installed reduction of the NLR (**Figure 4J**).

## Discussion

In this study, we explored the possible therapeutic value of the vagal nerve as an adjuvant treatment for RT. Precisely, we assessed whether transcutaneous VNS alters RT-induced immunosuppression in favor of more functional antitumor effector cells.

In mice we observed VNS-induced alterations in the immune response, such as the significant decrease in the splenic Treg population. Tregs are a subpopulation of CD4^+^ T helper cells capable of suppressing cytotoxic T cell responses (64). In many malignant diseases, immunosuppression by Tregs is known to play a central role in tumor immune escape and high Treg/CD8^+^ T cell ratios are correlated with a poor clinical outcome for NSCLC patients (65). Moreover, the induction of intratumoral Tregs is, in part, responsible for the development of resistance to anti-PD-1 therapy and PD-1^hi^ CD8^+^ T cells (66). Our results further show that both CCL1 and CCL22 are downregulated following VNS. In the murine LLC model, CCL22 has been identified as a chemokine involved in the recruitment of Tregs (67), while CCL1 was recently found to play a role in intratumoral accumulation of Tregs by driving the conversion of Tregs and enhancing their expressive function (55). In addition, CCL1 has been shown to stimulate the chemotaxis of neutrophils and as such possesses angiogenic properties, implicated in tumor growth, migration and invasion in NSCLC (68). In addition, VNS treatment improved the functional orientation of tumor infiltrated CD8^+^ T cells as evidenced by increased expression of IFN-γ and CD137. As several studies have shown the importance of CD137^+^ TILs and IFN-γ-producing cytotoxic CD8^+^ T cells for effective antitumor immunity (69, 70), the VNS-mediated stimulation of these CTLs could, next to the Treg decrease, also be responsible in part for the modest reduction in tumor burden upon VNS monotherapy.

Previously we showed that RT has several immune-suppressive consequences within the lung TME such as reduced numbers of matured antigen presenting cells and CTLs next to increased numbers of Tregs and MDSCs (22). While VNS in combination with RT did not significantly reduce any of these immunosuppressive alterations, it did result in a trend towards increased fractions of IFN-γ TILs. In accordance, we did not observe any significant changes in the serological levels of chemokines and cytokines between the RT + sham and RT + VNS treated groups. Notably, we further found that effector TILs of mice treated with RT + VNS expressed significant lower levels of PD-1 and thus are likely less susceptible to PD-L1-mediated immune suppression. Findings that are consistent with a study showing that sympathetic nerve denervation of tumors did not alter the number of CD4^+^ or CD8^+^ TILs but suppressed the expression of PD-1 on CD8^+^ TILs (71). In addition, VNS did significantly reduce the percentage of RT-induced splenic CD11b^+^ Ly6G^-^ Ly6C^hi^ monocytes. Of note, the addition of VNS to untreated (sham only) or RT treated mice, did not reduce tumor progression yet VNS did significantly augment the fraction of IFN-γ^+^ CD8^+^ T cells within the lung TME. Hence, VNS might be valuable to aid in tipping the balance from an overall suppressive to a more tumoricidal immune response especially in patients who qualify for RT in combination with immunotherapy. Yet more research will need to be performed to clarify the optimal doses and combination strategies. Taken together, our preclinical data do not support a slowed tumor growth upon VNS but a immunostimulatory effect on the intratumoral CD8^+^ T cells of RT treated lung tumor bearing mice.

Importantly, our murine results revealed that the impact of VNS treatment was limited to immune subsets in spleen and lung TME whereas no effects were observed in peripheral blood.

Using longitudinally collected blood samples from NSCLC patients, we identified several insignificant trends in immune cell shifts following VNS. Next to the elevation of NK cells and DCs, both linked to anti-tumor immunity, combinatorial ccRTCT + VNS resulted in lower levels of neutrophils compared to standard ccRTCT treated patients. In advanced NSCLC, a higher NLR has been shown to be a strong prognostic marker associated with an adverse prognosis (72), suggesting that our results are consistent with a potentially immunostimulatory effect of VNS.

So far, we were unable to identify significant differences in the human cohort. Apart from the lack of a large sample size, this might be linked to the large interpatient variation of our cohort regarding e.g tumor stage and histological subtype. Moreover, we want to stress that we were unable to show any significant changes in the evaluated blood samples derived from the NSCLC patient cohort as well as the VNS treated tumor-bearing mice. As we did observe significant immunological changes in the spleen and lung TME of VNS treated tumor-bearing mice, VNS seems more likely to affect the immune composition in spleen and lung TME than blood samples. An observation that is in line with a recent plea for more focus on patient-derived tissue than blood evaluation to unravel the immunological riddles that are at play during tumor progression and treatment (73). Hence, our study shows that future clinical evaluation of VNS should use a larger sample size, classify patients into different groups and aim to assess molecular and cellular changes at the tumor site.

## Conclusions

Our clinical and preclinical data suggest that VNS withholds potential to ameliorate local antitumor immunity as monotherapy as well as in combination with RT. Hence, we believe that assessing possible synergy between VNS and immunotherapies like ICIs and vaccination is the next step towards more VNS applications for cancer therapy. Yet, our data also reveal that larger clinical trials are indispensable to disclose the most optimal VNS stimulation parameters for its use as cancer monotherapy or as an adjuvant regimen. Altogether, our clinical data confirm that daily non-invasive transcutaneous VNS over period of 6 weeks in combination with (chemo)radiotherapy is feasible and well-tolerate by NSCLC patients, paving the way for more translation VNS studies in the immune-oncology field.

## Data Availability Statement

The raw data supporting the conclusions of this article will be made available by the authors, without undue reservation.

## Ethics Statement

The studies involving human participants were reviewed and approved by Commissie Medische Ethiek UZ Brussel. The patients/participants provided their written informed consent to participate in this study. The animal study was reviewed and approved by Ethics Committee of the Vrije Universiteit Brussel.

## Author Contributions

Conception and design, ER, CG, and YG. Development of methodology, ER and CG. Acquisition of data, ER and CG. Analysis and interpretation of data, ER, KD, and CG. Writing, review, and/or revision of the manuscript, ER, SD, HV, KD, TG, ED, LB, CC, LD, MDC, DL, JD, MDR, YG, and CG. Administrative, technical, or material support, ER, SD, HV, KD, TG, ED, and CG. All authors contributed to the article and approved the submitted version.

## Funding

This research was funded by research grants from Kom op Tegen Kanker (Stand up to Cancer), Fonds Wetenschappelijk Onderzoek Vlaanderen (FWO-V – grant number: 1515718N), Wetenschappelijk Fonds Willy Gepts and the Vrije Universiteit Brussel under the strategic research program scheme (grant SRP48).

## Conflict of Interest

The authors declare that the research was conducted in the absence of any commercial or financial relationships that could be construed as a potential conflict of interest.

## Publisher’s Note

All claims expressed in this article are solely those of the authors and do not necessarily represent those of their affiliated organizations, or those of the publisher, the editors and the reviewers. Any product that may be evaluated in this article, or claim that may be made by its manufacturer, is not guaranteed or endorsed by the publisher.
