# Supplementary material for: Transcutaneous Vagal Nerve Stimulation Alone or in Combination With Radiotherapy Stimulates Lung Tumor Infiltrating Lymphocytes But Fails to Suppress Tumor Growth
Source: Front Immunol. 2021 Dec 1;12:772555. doi: 10.3389/fimmu.2021.772555 (PMC8671299; doi:10.3389/fimmu.2021.772555)
Supplement: Supplementary file 1 [file DataSheet_1.docx]

**Supplementary Material**

**A**

**
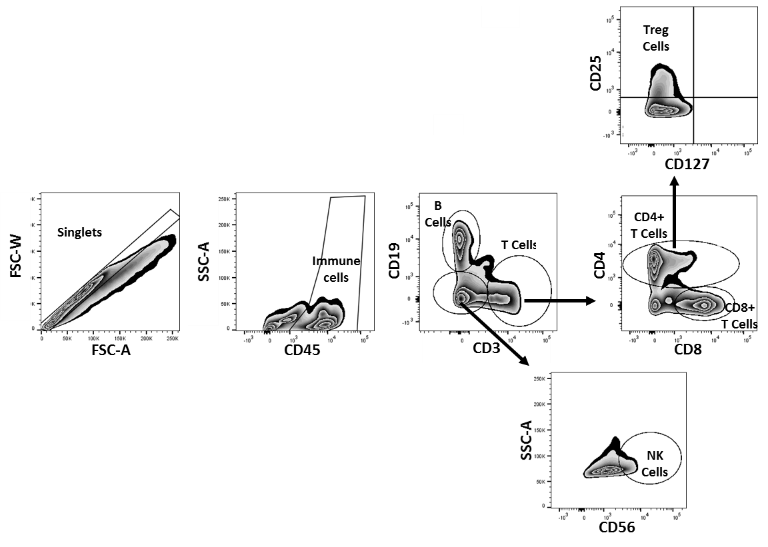
**

**B**


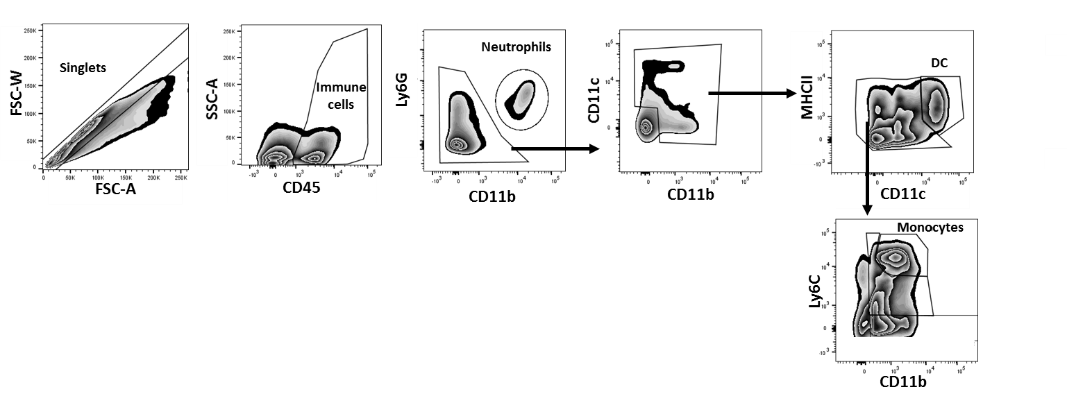


**C**

**
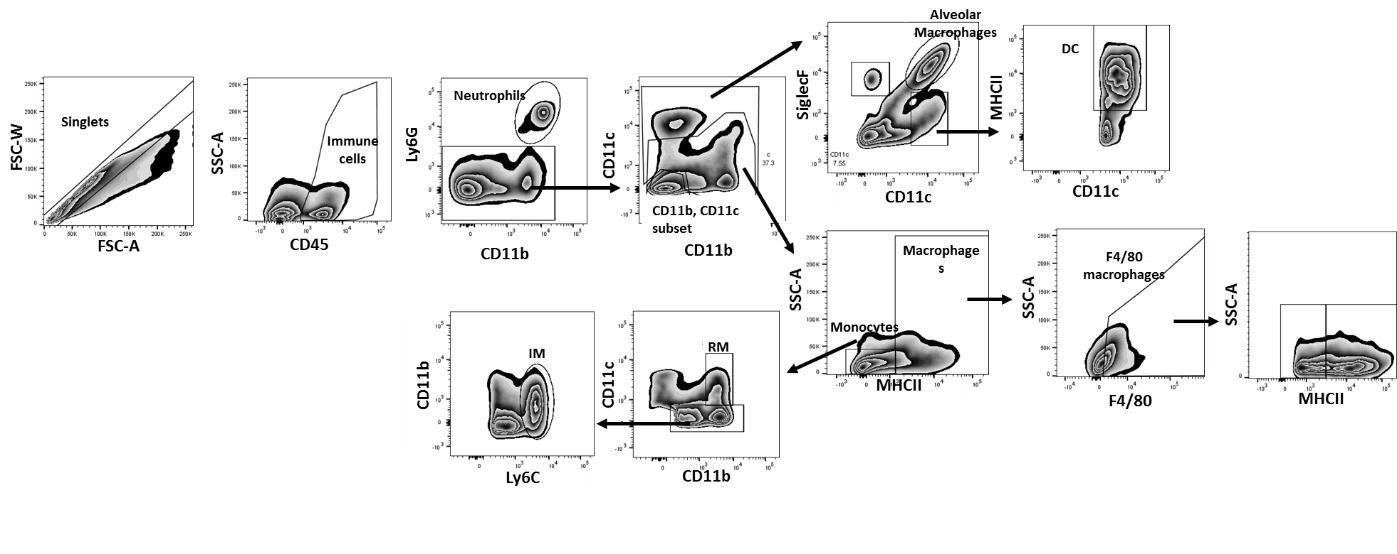
**

**Figure S1: Flow cytometry gating strategy used to identify lymphoid and myeloid cell subsets in LLC bearing mice.** **(A)** Representative dot plots to distinguish the following lymphoid subsets in blood, spleen and lung tumor tissue: CD19^+^ B cells, CD8^+^ CTLs, CD4^+^ T cells, CD56^+^ NK cells and CD25^+^/CD127^-^ Tregs. **(B)** Representative dot plots to distinguish the following myeloid subsets in blood and spleen: Ly6G^+^ neutrophils, CD11c^+^ DCs, conventional DCs (cDC) type 1 and 2 and plasmacytoid DC (pDC) in blood and spleen **(C)** Representative dot plots to distinguish the following myeloid subsets in lung tumor tissue: Ly6G^+^ neutrophils, Ly6C^+^ monocytes, eosinophils, SiglecF^+^ alveolar macrophages (AMs), CD11c^+^ DCs, CD103^+^ cDC1, CD11b^+^ cDC2, F4/80^+^ TAMs, MHCII^hi^ TAMs, MHCII^lo^ TAMs, CD11b^+^/MHCII^/^ Ly6C^+^inflammatory monocytes (IM) and Ly6C^-^ residential monocytes (RM).

**A**


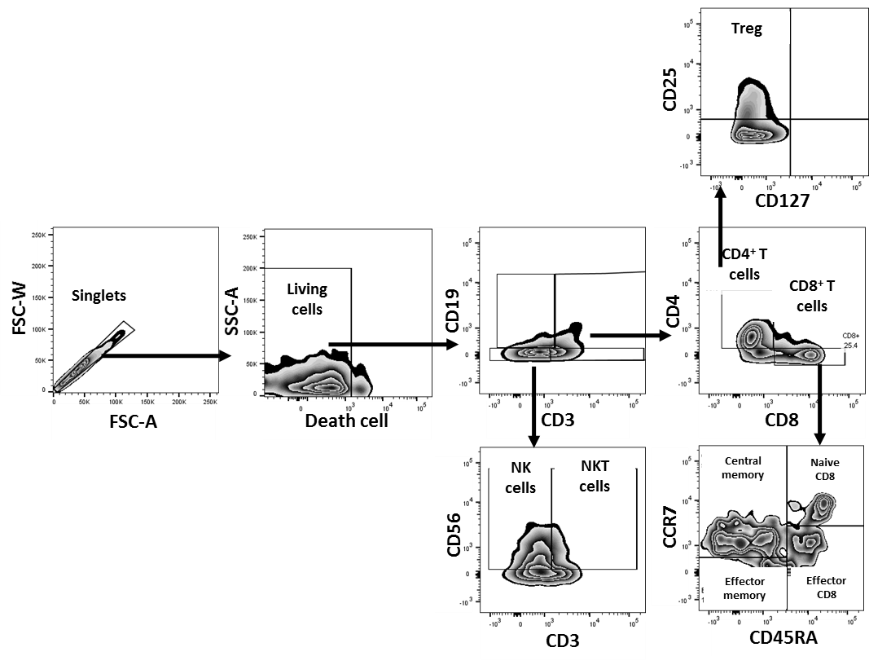


**B**

**
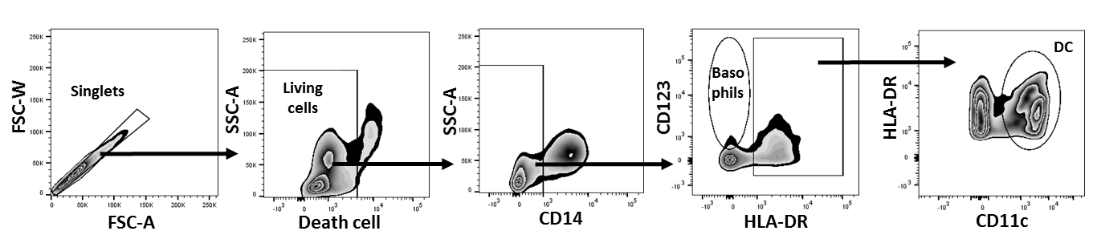
**

**C**

**
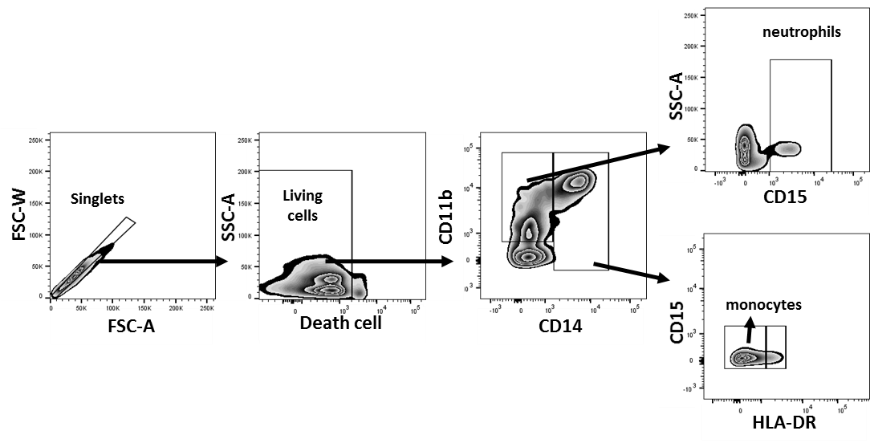
**

**Figure S2: Flow cytometry gating strategy used to identify circulating myeloid and lymphoid cell subsets in NSCLC patients.** Representative gating plots are shown for the following **(A)** lymphoid subsets: CD8^+^ CTLs, CD4^+^ T cells, NK cells, CD45RA^+^CCR7^+^ naïve CD8^+^ T, CD45RA^-^CCR7^+^ central memory CD8^+^ T, CD45RA^-^CCR7^-^ effector memory CD8^+^ T and CD45RA^+^CCR7^-^ effector CD8^+^ T; **(B)** DCs and **(C)** the myeloid subsets: monocytes (CD14^+^CD15^−^HLA-DR^lo/–^) and neutrophils (CD11b^+^CD14^−^CD15^+^).

**Supplementary Table 1: List of fluorescently labeled antibodies (murine markers)**

|  | Marker | Clone | Fluorochrome | Dilution | Vendor |
| --- | --- | --- | --- | --- | --- |
| Systemic  myeloid  panel | CD45 |  | BB515 | 1/200 | BD |
|  | CD11b | M1/70 | AF700 | 1/100 | Biolegend |
|  | Ly6G | IA8 | AF647 | 1/200 | BD |
|  | Ly6C |  | PE-Cy7 | 1/200 | BD |
|  | MHCII | M5/114.15.2 | PE/Dazzle 594 | 1/600 | Biolegend |
|  | CD11c | N418 | PerCPCy5.5 | 1/100 | Biolegend |
|  | B220 |  | APC-eF780 (Cy7) | 1/200 | BD |
|  | CD80 |  | BV510 | 1/200 | BD |
| Lung  Myeloid  panel | CD45.2 | 104 eBio | APC-eF780 (Cy7) | 1/100 | BD |
|  | CD11b | M1/70 | AF700 | 1/100 | Biolegend |
|  | Ly6G | IA8 | AF647 | 1/200 | BD |
|  | Ly6C |  | PECy7 | 1/200 | BD |
|  | MHCII | M5/114.15.2 | PE/Dazzle 594 | 1/600 | Biolegend |
|  | CD11c | N418 | AF488 | 1/200 | Biolegend |
|  | Siglec-F | E50-2440 | PE | 1/200 | BD |
|  | CD103 | M290 | PerCPCy5.5 | 1/100 | BD |
|  | F4/80 | BM8 | PerCPCy5.5 | 1/100 | Biolegend |
|  | CD86 | GL-1 | BV605 | 1/200 | Biolegend |
| Lymphoid  panel | CD45 |  | V450 | 1/100 | BD |
|  | CD3 | 145-2C11 | PerCPCy5.5 | 1/100 | Biolegend |
|  | CD4 | RM4-5 | AF700 | 1/200 | BD |
|  | CD8 | 53-6.7 | FITC | 1/200 | Biolegend |
|  | CD19 | 1D3 | AF647 | 1/100 | BD |
|  | CD25 | PC61 | BB515 | 1/200 | BD |
|  | CD127 | SB/199 | PE-CF594 | 1/200 | BD |
|  | PD1 | J43 | PE-Cy7 | 1/200 | Invitrogen |
|  | CD56 | BV510 | BV510 | 1/100 | BD |
| Functional panel | CD45 | 104 | APC-Cy7 | 1/200 | BD |
|  | CD3 | 17A2 | AF488 | 1/200 | Biolegend |
|  | CD8 | V450 | 53-6.7 | 1/200 | BD |
|  | CD4 | RM4-5 | AF700 | 1/200 | BD |
|  | CD137 | 17B5 | PE | 1/200 | Biolegend |
|  | IFN-γ | XMG1.2 | PECy7 | 1/200 | Biolegend |
|  | IL-2 | JES6-5H4 | AF647 | 1/200 | Biolegend |

**Supplementary Table 2: List of fluorescently labeled antibodies (Human markers)**

|  | Marker | Clone | Fluorochrome | Dilution | Vendor |
| --- | --- | --- | --- | --- | --- |
| DC  panel | Death cell |  | eF506 | 1/1000 |  |
|  | HLA-DR | L243 | PE-Cy7 | 1/100 | BD Pharmingen |
|  | CD123 | 9F5 | PE | 1/100 | BD |
|  | CD11c | B-ly6 | AF700 | 1/100 | BD |
|  | CD80 | 2D10.4 | PercpCy5.5 | 1/100 | eBioscience |
|  | CD40 | 5C3 | BV605 | 1/100 | BD OptiBuild |
|  | CD14 | M5E2 | BV421 | 1/100 | BD Bioscience |
|  | CD274 (PD-L1) | MIH1 | PE-CF594 | 1/100 | BD Bioscience |
| MDSC  panel | Death cell |  | 7AAD |  |  |
|  | CD14 | MOP9 | APC-H7 | 1/100 | BD Bioscience |
|  | CD11b | ICRF44 | PB | 1/100 | BD |
|  | HLA-DR | L243 | PE | 1/10 | BD |
|  | CD16 |  | VioGreen | 1/10 |  |
|  | CD15 | HI98 | FITC | 1/100 | BD Bioscience |
|  | CD274 (PD-L1) | MIH1 | PE-CF594 | 1/100 | BD Bioscience |
| Lymphocyte  panel | Death cell |  | 7AAD |  |  |
|  | CD3 | SK7 | BV605 | 1/100 | BD Bioscience |
|  | CD4 | RPA-T4 | APC-H7 | 1/100 | BD Pharmingen |
|  | CD8 | RPA-T8 | V500 | 1/100 | BD Horizon |
|  | CD19 | hib19 | BB515 | 1/100 | BD |
|  | CD56 | MEM-188 | PE-Cy7 | 1/100 | Biolegend |
|  | CD127 | HIL7RM21 | PE | 1/100 | BD |
|  | CD25 | BC96 | AF488 | 1/100 | Biolegend |
|  | CCR7 | G043H7 | APC | 1/25 | Biolegend |
|  | CD45RA | HI100 | BV421 | 1/100 | Biolegend |
| Functional  panel | Death cell |  | 7AAD |  |  |
|  | CD3 | SK7 | BV605 | 1/100 | BD Bioscience |
|  | CD8 | RPA-T8 | V500 | 1/100 | BD Horizon |
|  | CD107a | H4A3 | BV421 | 1/100 | BD |
|  | CD279 (PD-1) | MOPC-21 | PE | 1/100 | Biolegend |
|  | CD56 | MEM-188 | PE-Cy7 | 1/100 | Biolegend |
|  | IFN-g | B27 | FITC | 1/100 | BD Pharmingen |
|  | Granzyme B | GB11 | AF647 | 1/100 | BD Pharmingen |
